# Long-Term Clinical Relevance of Hyponatremia Identified During Acute Phase of Myocardial Infarction

**DOI:** 10.3390/jcm14030962

**Published:** 2025-02-03

**Authors:** Karolina Eliasz, Konrad Stępień, Maja Wojtylak, Oliwia Andrasz, Katarzyna Majka, Gabriela Mazurek, Grzegorz Horosin, Jakub Plizga, Karol Nowak, Krzysztof Krawczyk, Mateusz Podolec, Jadwiga Nessler, Jarosław Zalewski

**Affiliations:** 1 Department of Coronary Artery Disease and Heart Failure, Jagiellonian University Medical College, 31-202 Kraków, Poland; k.nowak.uj@gmail.com (K.N.); podolecmateusz@gmail.com (M.P.); jnessler@interia.pl (J.N.); j.zalewski@szpitaljp2.krakow.pl (J.Z.); 2 Student Research Group at Department of Coronary Artery Disease and Heart Failure, Jagiellonian University Medical College, 31-202 Kraków, Poland; maja.wojtylak@onet.pl (M.W.); oliwiaandrasz@gmail.com (O.A.); kasiamajka145@gmail.com (K.M.); gabrielaxmazurek@gmail.com (G.M.);; 3 4th Military Clinical Hospital, 53-114 Wrocław, Poland; jakubplizga7@gmail.com; 4 Department of Emergency Medicine, Faculty of Health Sciences, Jagiellonian University Medical College, 31-530 Kraków, Poland; krzysztof15.krawczyk@gmail.com; 5 Center for Innovative Medical Education, Jagiellonian University Medical College, 30-688 Kraków, Poland

**Keywords:** hyponatremia, acute myocardial infarction, prognosis

## Abstract

**Background/Objectives:** Hyponatremia is associated with increased mortality in the general hospital population. We sought to investigate whether hyponatremia affects the long-term survival of patients following a myocardial infarction (MI) in both ST-segment elevation (STEMI) and non-ST elevation (NSTEMI) presentations. **Methods**: In this study, 862 MI patients who were hospitalized between 2012 and 2017 were retrospectively followed-up within the median time period of 41.9 [28.2–73.5] months. All participants were assigned to a hyponatremic or normonatremic group with hyponatremia defined as a sodium level of less than 135 mEq/L on admission. **Results**: In the acute phase of an MI, hyponatremia was diagnosed in 31 (3.6%) patients. The patients with hyponatremia were less often male (38.7 vs. 70.4%, *p* < 0.001), and less frequently had Killip class I (63.3 vs. 80%) but more often had Killip class IV on admission (16.7 vs. 4.2%, *p* = 0.024) and more often had a history of impaired renal function (32.3 vs. 15.5%, *p* = 0.013) than those with normonatremia. Hyponatremic patients had higher troponin T levels on admission by 75.1% (*p* = 0.003), a higher isoenzyme MB of creatine kinase level by 34.4% (*p* = 0.006), and lower hemoglobin (by 8.5%, *p* = 0.001) levels as compared to the normonatremia group. Long-term mortality was significantly higher in the patients with hyponatremia versus normonatremia (18 [58.1%] vs. 243 [29.2%], log-rank *p* < 0.001). This was driven by differences in the NSTEMI population (65 vs. 30.5%, *p* < 0.001). By a Cox proportional hazard regression analysis, hyponatremia was associated with a higher long-term mortality (hazard ratio [HR] of 2.222, a 95% confidence interval [CI] of 1.309–3.773, and *p* = 0.003). **Conclusions**: Hyponatremia rarely identified in acute phase of MI was associated with higher long-term mortality, particularly in the NSTEMI population.

## 1. Introduction

Hyponatremia, defined as a sodium serum concentration below 135 mmol/L, is a quite common electrolyte abnormality, affecting up to 30% of hospitalized patients [1,2]. It may appear clinically significant in many conditions including dehydration and fluid loss, volume overload, hyperglycemia, nephrotic syndrome, cirrhosis, the syndrome of inappropriate antidiuretic hormone, psychogenic polydipsia, hypothyroidism, adrenal insufficiency, renal failure, a subarachnoid hemorrhage, or the use of loop diuretics in the treatment of patients with heart failure [2]. Hyponatremia has been considered an important marker of poor prognosis in various clinical settings including heart failure [3,4,5], stroke [6], chronic kidney disease [7], and many other conditions [8].

Hyponatremia was found to be a significant predictor of mortality in patients with an acute myocardial infarction (MI) but the disparities in its prognostic value in specific MI presentations remain not well established. According to the results of a meta-analysis conducted by Ma et al., numerous studies revealed significantly higher 30-day mortality rates in acute coronary syndrome patients with hyponatremia (with a relative risk [RR] of 2.18, a 95% confidence interval [CI] of 1.96 to 2.42, and *p* < 0.001) [9]. In addition, similar outcomes were obtained for long-term mortality during the up to 18-year follow-ups (hazard ratio [HR] of 1.74, a 95% CI of 1.56–1.942, and *p* < 0.001) [9]. According to the current knowledge, an MI is divided into two main subtypes: an ST segment elevation myocardial infarction (STEMI) and a non-ST segment elevation myocardial infarction (NSTEMI) [10]. These two groups of patients differ in clinical manifestations, treatment, and short- and long-term prognoses [11,12,13,14,15]. In several studies, it has been proven that hyponatremia is an important prognostic factor in STEMI and MI patients without specifications about its presentation. Sanchez et al. found that the differences in the prevalence of NSTEMI and STEMI presentation in hyponatremic patients were not statistically significant; however, hyponatremia on admission and during hospitalization was associated with increased mortality, regardless of MI type [9,16,17]. Interestingly, the non-osmotic release of arginine vasopressin with subsequent dilutional hyponatremia in the patients with STEMI complicated by symptoms of congestive heart failure was observed [17]. Moreover, a significantly increased 30-day mortality in the NSTEMI patients with hyponatremia (13.8 vs. 7.5%) was found by Singla et al., suggesting that hyponatremia may also be an unfavorable prognostic factor in this group of patients [18].

The aim of this study was to identify the association between as measured on admission serum hyponatremia and the long-term mortality rate of the cohort of Polish MI patients with STEMI and NSTEMI.

## 2. Materials and Methods

According to the European Society of Cardiology (ESC) guidelines, an MI is defined as myocardial cell death due to prolonged ischemia. In our current study, we focused on patients with type 1 MI, which are caused by atherothrombotic coronary artery disease (CAD). We enrolled 862 type 1 MI patients who were hospitalized between 2012 and 2017. The diagnosis of an MI was based on the clinical presentation and the detection of a rise and/or fall in cTn values, with at least one value above the 99th percentile URL. The mandatory inclusion criterion was a performed coronary angiography. Among these patients, based on the ECG records, we distinguish two types—STEMI and NSTEMI. STEMI presentation was diagnosed when the new ST-segment elevation at the J-point in at least two contiguous leads amounted to ≥2.5 mm in men <40 years of age, ≥2 mm in men ≥40 years of age, or ≥1.5 mm in women regardless of age for leads V2–V3 and/or ≥1 mm in the other leads. STEMI presentation also included patients presenting with a new left or right bundle branch block. No changes in ECG or other than those mentioned above enhanced for NSTEMI diagnosis. All of the patients underwent an assessment of their on-admission electrolyte levels as a standard procedure. We incorporated patients whose sodium level did not exceed 145 mEq/L. The baseline characteristics included anthropometric data, risk factors, comorbidities, laboratory results obtained on admission, data regarding the course of hospitalization, and medications on discharge [19]. Hyponatremia was defined as a serum sodium level of less than 135 mEq/L. All patients were assigned to the hypo- or normonatremia group. Renal function was assessed with the Cockcroft–Gault formula and a creatinine clearance level below 60 mL/min indicated impaired renal function.

In all patients, immediately after admission, a coronary angiography was performed with a subsequent revascularization if needed [20]. All coronary angiographics afterwards were thoroughly evaluated by two blinded physicians to determine infarct-related artery (IRA) patency and to assess critical lesions along with the results of the primary percutaneous coronary intervention (PCI). In case of a lack of obstructive lesions, narrowing the epicardial coronary segments by more than 50% during an angiography myocardial infarction with non-obstructive coronary arteries (MINOCA) was recognized [20]. Every patient underwent a two-dimensional transthoracic echocardiography at rest between the 2nd and 4th day of hospitalization after the stabilization of the patients to assess left ventricular ejection fraction (LVEF) with the Simpson’s method [19].

Data on the long-term outcomes, all-cause mortality rate, and its date were obtained from the Polish National Death Registry [21]. In our study, the long-term observation period was a median time of 41.9 [28.2–73.5] months. None of the patients were lost to follow-up. The study protocol complied with the Declaration of Helsinki and was approved by the relevant local Ethics Committee. Informed consent was provided by all the patients participating in this study.

### Statistical Analysis

A statistical analysis was performed using IBM SPSS Statistics Version 26.0 (IBM Corp., Armonk, NY, USA). Continuous variables were first checked for normal distribution using the Shapiro–Wilk test. Due to the vast majority of continuous variables being characterized by a non-normal distribution, they were expressed as medians (interquartile range), and categorical variables as numbers (percentages). The differences in continuous variables were compared by a Student’s *t*-test or Mann–Whitney U test if the distribution was normal or different than normal, respectively [19]. Categorical variables were analyzed with the chi-square test or Fisher’s exact test. Kaplan–Meier curves for overall mortality were constructed to estimate the survival rates in the overall population as well as in the NSTEMI and STEMI subgroups. A log-rank test was performed to assess the differences in survival rates between the studied groups [19]. A multivariable linear regression was performed to find the independent determinants of baseline sodium levels. Finally, all independent variables potentially associated with an outcome were included in the Cox proportional hazard regression model to determine the independent predictors of long-term mortality. A two-sided *p*-value of less than 0.05 was considered statistically significant.

## 3. Results

### 3.1. Clinical Characteristics

**Figure 1:**
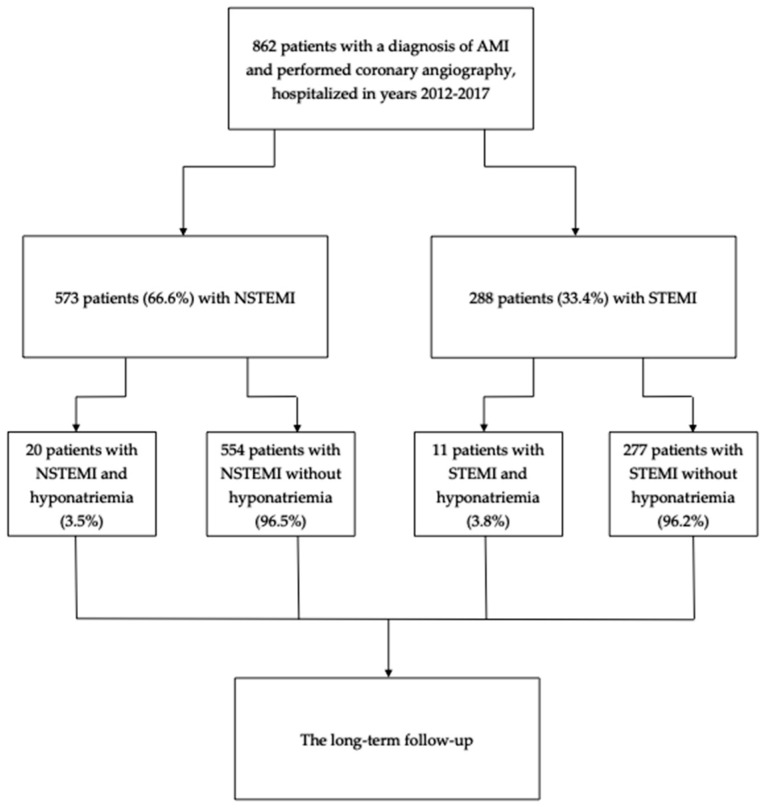
The study flow-chart.

Of the 862 analyzed patients, hyponatremia was diagnosed in 31 participants (3.6%) (Figure 1). The sodium levels of those patients were significantly lower (132 [130–133] vs. 140 [138–142] mEq/L, *p* < 0.001) than in the normonatremic patients.

The hyponatremic group had a higher proportion of women in comparison to the group of normonatremic patients (61.3 vs. 29.6%, *p* < 0.001) (Table 1). Significant differences in age, history of stroke, a prior MI incidence, prior revascularization, a left ventricular ejection fraction, as well as clinical presentation and medications on discharge between the compared groups were not identified. Both groups were also similar in terms of the majority of cardiovascular risk factors, except for impaired renal function which occurred more frequently in the hyponatremic patients (32.3 vs. 15.5%, *p* = 0.013). In addition, the patients without hyponatremia were characterized by less severe clinical symptoms on admission. Patients with Killip class I were more frequently found in the normo- versus hyponatremic patients (80 vs. 63.3%) while those with Killip class IV appeared less frequently (4.2 vs. 16.7%, *p* = 0.024).

Some differences in the laboratory parameters on admission were identified (Table 1). Myocardial necrotic markers including troponin T (by 75.1%, *p* = 0.003) and the isoenzyme MB of creatine kinase (by 34.4%, *p* = 0.006) levels were higher in the hyponatremic group. In this group, higher levels of white blood cells, glucose, and creatinine were also found. In contrast, lower levels of hemoglobin, hematocrit, and glomerular filtration rate were observed in that group (Table 1).

### 3.2. Angiography and Revascularization Strategy

The angiographic analysis did not reveal significant differences in the distribution of IRA and the frequency of an MINOCA diagnosis (Table 2). There was also a lack of differences in the revascularization strategy. The most performed revascularization therapy in hypo- and normonatremic groups was a primary PCI (87.1 and 81.1%, respectively) (Table 2).

### 3.3. Long-Term Mortality

**Figure 2:**
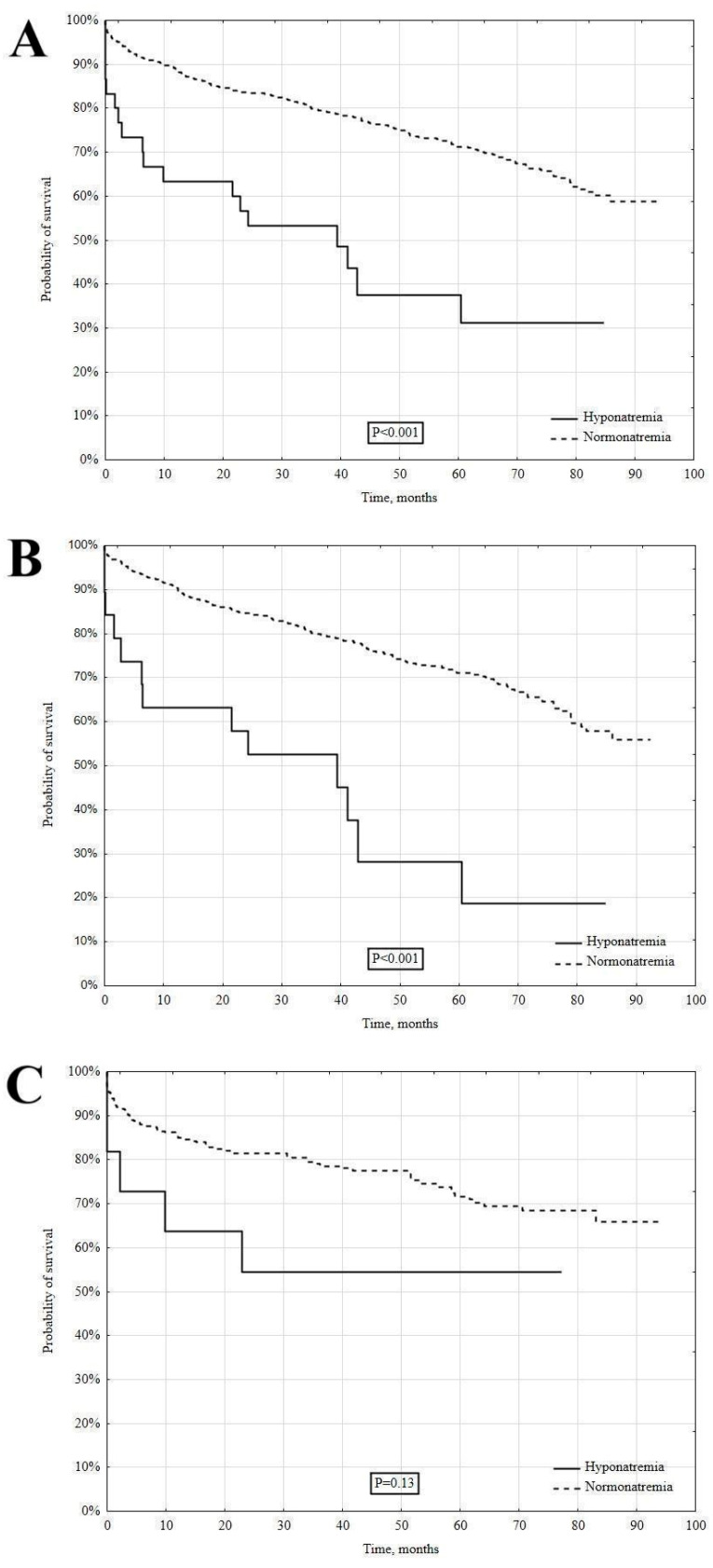
The long-term survival in hypo- and normonatremic patients. Total group (**A**), NSTEMI (**B**), and STEMI (**C**).

During the median time of 41.9 [28.2–73.5] months, the long-term all-cause mortality rate was significantly higher in the patients with hyponatremia versus normonatremia (18 [58.1%] vs. 243 [29.2%], log-rank *p* < 0.001) (Figure 2A). In the analysis of MI subtypes, hyponatremia in the NSTEMI patients was associated with a significantly higher long-term mortality (65 vs. 30.5%, log-rank *p* < 0.001) (Figure 2B). In contrast, a similar difference was not observed in the STEMI patients (45.5 vs. 26.7%, log-rank *p* = 0.13) (Figure 2C).

As determined by multivariable linear regression, there are several factors associated with the baseline sodium levels of the whole studied group (R^2^ = 0.133, *p* < 0.001, Table 3). As has been shown, lower hematocrit levels were associated with lower sodium levels. In turn, an inverse relationship was observed for creatinine, the isoenzyme MB of creatinine kinase, and glucose levels (Table 3).

As determined by the Cox proportional hazard regression analysis, the independent determinants of mortality were a higher age, a lower LVEF, a higher Killip class, and lower hemoglobin levels (Table 4). Apart from these, a hyponatremia diagnosis was also an independent predictor of long-term all-cause mortality (hazard ratio [HR] of 2.222, a 95% confidence interval [CI] of 1.309–3.773, and *p* = 0.003) (Table 4).

## 4. Discussion

This study demonstrated that hyponatremia in MI patients is associated with a significantly higher long-term mortality, particularly in NSTEMI patients. The hyponatremic patients presented more severe clinical symptoms on admission, including a worse Killip class and higher levels of troponin T and the isoenzyme MB of creatinine kinase. Despite similar coronary revascularization treatments between groups, the patients with hyponatremia had unfavorable outcomes during the median follow-up period of almost 5 years. Finally, the multivariable model confirmed hyponatremia as an independent predictor, doubling the mortality risk when compared with the normonatremic patients.

The proper identifying of high-risk patients may mean opting for more aggressive management, such as early invasive strategies or enhanced pharmacological interventions. One of the major causes of hyponatremia in AMI patients is fluid overload due to progressive heart failure. Knowing this, practicians might be more cautious with fluid administration or consider the intensification of heart failure treatment. Patients with hyponatremia may need to be admitted to an intensive care unit (ICU) for closer monitoring of their fluid and electrolyte statuses, as well as their cardiac and renal functions. This enables a timely intervention if complications arise. It is also an indication of the need to optimize the patient’s pharmacotherapy (especially those diagnosed with heart failure) upon discharge. Hyponatremia can indicate more severe underlying organ damage. Therefore, post-AMI management may require a more comprehensive approach, including rehabilitation, a closer follow-up, and monitoring for heart failure progression or recurrent events.

It is interesting that the prevalence of hyponatremia in our MI population was almost 10-times lower than the estimated rate of low sodium levels in the general hospitalized patients [1,2]. We find it difficult to explain, considering that acute MI occurs more often in the group of older patients as in our study, and MI represents an acute condition, in which we expected lower sodium levels. In our analysis, as has been proven in previous studies, hyponatremia occurs more frequently in female patients [22]. The underlying mechanism of it has not been fully understood. The exposure to estrogen and progesterone which changes during the menopause period affects arginine vasopressin (AVP) regulation in women [23]. It was also hypothesized that the distribution of renal transporters along the nephron is different between men and women. The greater amount of renal Na^+^ transporters in the proximal tubules in females may be connected with their need for adaptation to increase fluid retention during pregnancy and lactation [24]. More research is required to understand the sex differences and their clinical implications.

In the existing literature, there are some data regarding the influence of hyponatremia on prognosis in acute MI patients. In the study by Choi et al., hyponatremia was found to have a significant influence on long-term mortality only when present upon discharge [25]. The results of the Goldberg et al. study revealed findings in line with ours; however, only patients with STEMI were assessed [26]. In this study, hyponatremia as measured on admission increased mortality in the long-term, particularly within the almost 5-year follow-up period. In addition, hyponatremia remained an independent predictor of long-term all-cause mortality (HR of 2.0, 95% CI of 1.3–3.2, and *p* = 0.002) [26]. Consistent results were received by Havránek et al. during the mean follow-up period of 39 months [27]. The STEMI patients with hyponatremia on admission had a lower LVEF, an elevated Killip class, and a higher death rate when compared with the normonatremic group (34.7% vs. 20.5%, *p* = 0.02) [27]. Furthermore, Burkhardt et al. analyzed the influence of hyponatremia on STEMI and NSTEMI patients separately, discovering that there was no significant difference in mortality between the different MI types. Both groups with hyponatremia had a lower long-term survival rate than the normonatremic participants [28].

The mechanisms dependent on hyponatremia and responsible for a worse prognosis in acute MI patients can be associated with the release of vasopressin, and the activation of the sympathetic and renin– angiotensin–aldosterone systems [29]. According to the current knowledge, these mechanisms lead to peripheral vasoconstriction and myocardial hypertrophy [30,31]. Hyponatremia also affects the myocardium directly by altering the action of the sodium–calcium pump in cardiomyocytes, which leads to a calcium overload and may induce myocardial oedema, contractile dysfunction, and coronary vasoconstriction [32]. Moreover, it enhances reactive oxygen species production which increases myocardial injury. In this context, hyponatremia can worsen cardiac vulnerability to ischemia and reperfusion injury in the acute phase of an MI [33].

The relatively high mortality rate observed in the MI patients—both with hypo- and normonatremia—who were admitted to our clinic in 2012–2017 can be partly attributed to factors such as delayed diagnoses due to the lack of sufficient public education on cardiovascular diseases or limited access to specialized care, and suboptimal management practices. Additionally, this period predates the introduction of key treatments for heart failure management, such as flozins (SGLT2 inhibitors) and sacubitril/valsartan, which have since become integral to improving outcomes. The lack of these advanced therapeutic options likely contributed to the poor prognosis of many MI patients, particularly those with heart failure and a reduced ejection fraction or other comorbidities. However, in more recent years, significant improvements have been made in patient care following an MI, with the development of numerous monitoring programs, such as the “KOS-MI” initiative, which tracks and supports patients after their hospitalization [15,34]. These programs have proven to be highly effective in improving long-term outcomes and survival rates, demonstrating the positive impact of more structured follow-up care and early intervention [15,34]. As a result, the overall prognosis for MI patients in Poland has markedly improved in recent years.

Our study has several limitations. Firstly, the analyzed patients were recruited in a single center; therefore, the results possibly do not represent the whole Polish population adequately. Secondly, patients’ sodium levels were assessed only on admission and were not evaluated throughout their hospital stay, thus the comparison of mortality depending on natremia fluctuations could not be performed. We also did not consider all the possible causes of hyponatremia and their impact on the results of our study. Moreover, we did not classify them as primary and secondary. Finally, we were not able to provide the causes of patients’ death due to the limitations of the analyzed death registry.

## 5. Conclusions

Although relatively rare, hyponatremia in the acute phase of MI remains an independent predictor of increased long-term all-cause mortality, particularly with NSTEMI presentation. For clinicians, it is important to consider electrolyte imbalance when assessing a patient’s prognosis and possibly adjusting a treatment regimen. Further studies concerning this relationship need to be performed with a representative number of participants to understand the underlying mechanisms and plan the optimal management of the condition to ensure favorable long-term clinical outcomes as frequently as possible.

## Figures and Tables

**Table 1:** Characteristics of the studied patients.

	Hyponatremia N = 31	Non-Hyponatremia N = 831	*p*-Value
Male gender	12 (38.7)	585 (70.4)	<0.001
Age, years	74 (60–81)	68 (60–78)	0.10
Body mass index, kg/m^2^	27.3 (23.5–31.1)	27.6 (24.9–30.9)	0.32
Diabetes mellitus	15 (50.0)	309 (37.3)	0.16
Hypertension	24 (80.0)	731 (88.2)	0.18
Dyslipidemia	24 (80.0)	701 (84.6)	0.50
Impaired renal function	10 (32.3)	129 (15.5)	0.013
Active smoking	5 (16.7)	198 (23.9)	0.36
Chronic heart failure	11 (36.7)	276 (33.3)	0.70
Peripheral arterial disease	4 (13.3)	84 (10.1)	0.57
Prior stroke	4 (13.3)	53 (6.4)	0.13
Prior myocardial infarction	11 (36.7)	235 (28.4)	0.32
Prior revascularization			
Percutaneous coronary intervention	7 (23.3)	150 (18.1)	0.45
Coronary artery bypass surgery	0 (0.0)	34 (4.1)	
			
Both percutaneous coronary intervention and coronary artery bypass surgery	0 (0.0)	27 (3.3)	
Killip class on admission:			
I	19 (63.3)	665 (80.0)	0.024
II	5 (16.7)	101 (12.2)	
III	1 (3.3)	29 (3.5)	
IV	5 (16.7)	35 (4.2)	
Left ventricular ejection fraction	45 (30–55)	50 (40–55)	0.14
			
Clinical presentation			
NSTEMI	20 (64.5)	554 (66.7)	0.80
STEMI	11 (35.5)	277 (33.3)	
Laboratory tests on admission			
Troponin T, ng/mL	0.426 (0.043–1.88)	0.106 (0.029–0.382)	0.003
Creatine kinase, IU/L	239 (159–711)	177 (106–368)	0.07
Isoenzyme MB of creatine kinase, IU/L	32 (22–92)	21 (15–40)	0.006
Sodium, mEq/L	132 (130–133)	140 (138–142)	<0.001
Potassium, mEq/L	4.2 (3.8–4.8)	4.1 (3.8–4.5)	0.89
Hemoglobin, g/dL	12.9 (11.5–14.1)	14.1 (12.9–15)	0.001
Hematocrit, %	38 (34–42)	42 (39–45)	<0.001
White blood cells, ×10^3^/µL	10.7 (8.6–15)	9.2 (7.4–11.7)	0.014
Platelet count, ×10^3^/µL	248 (179–286)	221 (184–270)	0.46
Glucose, mmol/L	9.9 (5.7–14.9)	6.8 (5.8–8.9)	0.012
Creatinine, µmol/L	112 (80–140)	88 (76–103)	0.007
Glomerular filtration rate, mL/min	47 (34–79)	71 (58–86)	<0.001
Total cholesterol, mmol/L	4.1 (3.2–5.6)	4.4 (3.6–5.3)	0.31
LDL cholesterol, mmol/L	2.2 (1.6–3.2)	2.6 (1.7–3.4)	0.64
HDL cholesterol, mmol/L	1.1 (0.9–1.5)	1.3 (1–1.7)	0.23
Triglycerides, mmol	1.2 (0.8–2)	1.3 (0.9–1.7)	0.79
Medications on discharge			
Aspirin	31 (100.0)	831 (100.0)	1.00
P2Y12 inhibitor	30 (96.8)	828 (99.6)	0.83
ACE-I	21 (72.4)	669 (80.6)	0.28
Beta-adrenolytic	27 (93.1)	743 (89.5)	0.53
Statin	27 (90.0)	775 (93.4)	0.47
Loop diuretic	9 (29.0)	194 (23.3)	0.67

Abbreviations: data are shown as the median (interquartile range) or a number (percentage), ACE-I: angiotensin-converting enzyme inhibitor, HDL: high-density lipoprotein, LDL: low-density lipoprotein, NSTEMI: non-ST-segment elevation myocardial infarction, and STEMI: ST-segment elevation myocardial infarction.

**Table 2:** Angiography and revascularization in-the studied-groups.

	Hyponatremia N = 31	Non-Hyponatremia N = 831	*p*-Value
Infarct-related artery:			
Left main	3 (9.7)	31 (3.7)	0.10
Left anterior descending/diagonal branch	10 (32.3)	269 (34.4)	
Left circumflex/marginal branch	4 (12.9)	175 (21.1)	
Right coronary artery	14 (45.2)	279 (33.6)	
Undetermined	0 (0.0)	77 (9.3)	
Diagnosis of MINOCA	0 (0.0)	66 (7.9)	0.10
Treatment:			
Primary percutaneous coronary intervention	27 (87.1)	674 (81.1)	0.56
Coronary artery bypass surgery	0 (0.0)	22 (2.7)	
Conservative	4 (12.9)	135 (16.3)	

Abbreviations: data are shown as a number (percentage), and MINOCA: myocardial infarction with nonobstructive coronary arteries.

**Table 3:** The independent factors associated with baseline serum sodium level.

Independent Variable		Univariable Model		Multivariable Model		
	Beta	95% CI for Beta	*p*-Value	Beta	95% CI for Beta	*p*-Value
Sex, female vs. male	0.094	0.028–0.160	0.006	0.061	−0.014–0.124	0.117
Hematocrit, per 1%	0.181	0.116–0.248	<0.001	0.138	0.070–0.206	<0.001
Creatinine, per 1 µmol/L	−0.129	−0.204–−0.074	<0.001	−0.104	−0.173–−0.035	0.002
Isoenzyme MB of creatine kinase, per 1 IU/L	−0.147	−0.208–−0.083	<0.001	−0.111	−0.178–−0.044	<0.001
Glucose, per 1 mmol/L	−0.289	−0.354–−0.223	<0.001	−0.248	−0.314–−0.181	<0.001

Abbreviations: CI: confidence interval.

**Table 4:** The independent predictors of long-term mortality.

Independent Variable		Univariable Model		Multivariable Model		
	HR	95% CI for HR	*p*-Value	HR	95% CI for HR	*p*-Value
Age, per 1 year	1.058	1.046–1.071	<0.001	1.046	1.033–1.059	<0.001
Impaired renal function, no vs. yes	2.616	2.006–3.412	<0.001	1.307	0.974–1.752	0.074
LVEF, per 1%	0.948	0.939–0.957	<0.001	0.967	0.956–0.977	<0.001
Killip class, per 1 class	2.271	2.019–2.555	<0.001	1.677	1.426–1.971	<0.001
Hyponatremia, no vs. yes	2.734	1.693–4.415	<0.001	2.222	1.309–3.773	0.003
Hemoglobin, per 1 g/dL	0.787	0.751–0.826	<0.001	0.825	0.778–0.876	<0.001

Abbreviations: CI: confidence interval, HR: hazard ratio, and LVEF: left ventricular ejection fraction.

## Data Availability

The original contributions presented in this study are included in the article. Further inquiries can be directed to the corresponding authors.
